# Using Machine Learning and Molecular Docking to Leverage Urease Inhibition Data for Virtual Screening

**DOI:** 10.3390/ijms24098180

**Published:** 2023-05-03

**Authors:** Natália Aniceto, Tânia S. Albuquerque, Vasco D. B. Bonifácio, Rita C. Guedes, Nuno Martinho

**Affiliations:** 1 Research Institute for Medicines (iMed.ULisboa), Faculty of Pharmacy, Universidade de Lisboa, 1649-003 Lisbon, Portugal; 2 Department of Pharmaceutical Sciences and Medicines, Faculty of Pharmacy, Universidade de Lisboa, 1649-003 Lisbon, Portugal; 3 iBB—Institute for Bioengineering and Biosciences, and Associate Laboratory i4HB—Institute for Health and Bioeconomy at Instituto Superior Técnico, Universidade de Lisboa, Av. Rovisco Pais, 1049-001 Lisboa, Portugal; 4 Bioengineering Department, Instituto Superior Técnico, Universidade de Lisboa, Av. Rovisco Pais, 1049-001 Lisboa, Portugal

**Keywords:** urease, machine learning, random forest, protein–ligand interactions, QSAR, *H. pylori*, jack bean urease

## Abstract

Urease is a metalloenzyme that catalyzes the hydrolysis of urea, and its modulation has an important role in both the agricultural and medical industry. Even though numerous molecules have been tested against ureases of different species, their clinical translation has been limited due to chemical and metabolic stability as well as side effects. Therefore, screening new compounds against urease would be of interest in part due to rising concerns regarding antibiotic resistance. In this work, we collected and curated a diverse set of 2640 publicly available small-molecule inhibitors of jack bean urease and developed a classifier using a random forest machine learning method with high predictive performance. In addition, the physicochemical features of compounds were paired with molecular docking and protein–ligand fingerprint analysis to gather insight into the current activity landscape. We observed that the docking score could not differentiate active from inactive compounds within each chemical family, but scores were correlated with compound activity when all compounds were considered. Additionally, a decision tree model was built based on 2D and 3D Morgan fingerprints to mine patterns of the known active-class compounds. The final machine learning model showed good prediction performance against the test set (81% and 77% precision for active and inactive compounds, respectively). Finally, this model was employed, as a proof-of-concept, on an in-house library to predict new hits that were then tested against urease and found to be active. This is, to date, the largest, most diverse dataset of compounds used to develop predictive in silico models. Overall, the results highlight the usefulness of using machine learning classifiers and molecular docking to predict novel urease inhibitors.

## 1. Introduction

Urease is a metalloenzyme conserved in several organisms, including plants, fungi and bacteria, that catalyzes the rapid hydrolytic decomposition of urea into ammonia and carbamate [1]. This reaction is key in the global nitrogen cycle of organisms, and urease is also an important pathogenic factor that confers resistance to bacteria such as *H. pylori* and *P. mirabilis*. In particular, the former relies on urease for survival in the highly acidic environment of the stomach. Due to the role of ureases in pathogen survival, their inhibition has shown tremendous potential both for agricultural and medical applications as well as in the development of sensors.

A wide array of small molecules of both synthetic and natural origin have been tested against ureases of different species to modulate their activity [2,3,4]. Generally, inhibition is achieved through targeting the conserved bimetallic nickel ions using substrate-like compounds, transition-state analogues and metal chelating moieties. However, other strategies that involve covalent modification of an important cysteine that is essential for the catalytic mechanism [5,6], non-competitive inhibition, as well as other non-specific mechanisms (e.g., precipitation, denaturation), have shown promising results [6]. However, so far, these compounds have had limited translation to the clinical setting due to issues of cost, potency, solubility, and chemical and metabolic stability as well as toxicity due to promiscuous binding to other enzymes. Recently, high-throughput screening of FDA-approved drugs as a re-purposing strategy was carried out to find inhibitors of urease, but the most potent compounds were found to be anti-cancer drugs and consequently not appropriate for this application [7]. Therefore, there is still much interest and potential in developing new inhibitors, but the de novo design of lead molecules is still a difficult challenge.

Data-driven design of new compounds in medicinal chemistry is essential at various stages of lead development. In particular, searching for novel compounds that display activity greatly benefits from insights gained from other classes tested. Recently, we reported on the chemical space of urease inhibitors and found a diverse range of scaffolds and key features associated with activity [4]. Several in silico approaches, including simple quantitative structure–activity relationship (QSAR) models, molecular docking and machine learning methods, have been applied to successfully predict urease inhibition activity and to screen new compounds [8,9]. However, these studies generally do not combine different approaches (e.g., docking score, protein-ligand interactions and machine learning) for predictive analysis and use a limited number of total compounds and classes of compounds and, therefore, lack the structural features that contribute to activity. As a result, constructing a predictive model with wide applicability and high performance has not been done yet. Furthermore, the models in these studies were built with limited complexity (i.e., using only one type of feature/descriptor), which potentially compromises capturing more complex patterns that define inhibitors and may, in turn, compromise the model’s performance when screening new compounds. Moreover, to date, there is no study that carried out molecular docking on all urease inhibitors using the same protocol and enzyme. Therefore, in order to further expand on our previous analysis [4], we applied computational methods of molecular docking and machine learning to identify scaffolds with activity. Since jack bean urease is typically used as a surrogate to test the inhibitory activity against other species as its active site is well conserved, this enzyme was selected due to the higher number of compounds tested against it. To the best of our knowledge, this constitutes the largest dataset of urease inhibitors and, therefore, this work is the most comprehensive and most thoroughly validated modelling effort for this enzyme. Furthermore, we highlighted potential residues to target when designing novel urease inhibitors. Finally, we also reported on the use of machine learning algorithms to design a transparent classifier based on molecular descriptors and features derived from molecular docking and how they can be useful in screening new compounds, as well as their limitations. Additionally, we used this in consensus with the docking scores produced by a molecular docking model to predict new active compounds. After validation on two external datasets gathered after the model has been built, both models were used, as a proof-of-concept, to screen an in-house set of compounds tested against jack bean urease.

## 2. Results and Discussion

### 2.1. Active and Inactive Classes Show Similar Key Chemical Properties

A diverse collection of scaffolds has been tested against ureases of different species, but the majority of studies have used jack bean urease as a surrogate due to its relatively low cost and commercial availability. Jack bean urease also shows generally good translation into the other species due to the highly conserved active site [4]. Nevertheless, to avoid noise from aggregating data from different species when training our model, we chose to restrict our data to jack bean urease exclusively due to this organism having the largest set of activity data.

An initial dataset of 1614 active and inactive compounds (~1:1 ratio) tested against jack bean urease was assembled (Table 1). Considering that compounds are assigned to either activity class according to their ratio of activity against the control in the corresponding assay, it is worth noting that the majority of the inactive compounds showed a maximum activity ratio of 2 (i.e., twice the control activity). This dataset was then split into two additional subsets: training and test set (75%:25%) (Table 1) using clustered sampling (see methods) [4], to avoid entire clusters being left out from training. An external validation set was also assembled in order to extend validation (external validation set). Additionally, while building the predictive model, newly reported compounds were continuously being collected, and these were used as a temporal dataset to assess the performance throughout time with newly reported scaffolds. All datasets showed a low Tanimoto mean value (0.13–0.15), and therefore, a high diversity in each dataset was identified (Table 1). For all datasets, the majority of both active and inactive compounds had similar distributions of molecular weight and number of heavy atoms, lipophilicity (MolLogP) and topological polar surface area (TPSA) (Figure S1).

### 2.2. Docking Score Is Predictive of Urease Inhibition

Having an understanding of the key structural underpinnings that drive ligand binding at the active site is an important step in the design of novel inhibitors. In this regard, docking is a very important tool, as it generally produces binding poses that can then be analyzed to determine key interactions that correlate with activity and can thus be used for screening. We carried out molecular docking calculations for all datasets using the X-ray structure of jack bean urease in the bound state (PDB 4H9M). We first validated the docking protocol with self-docking of acetohydroxamic acid and observed a high performance in predicting the crystallographic ligand pose (root mean square deviation < 1 Å). This protocol was then extended to the rest of the compounds. Since docking generates a number of energetically acceptable binding modes (poses), we compared the first docking pose and the top five poses and did not observe any significant differences in terms of docking scores between all poses, which varied mostly within 0.5 kcal/mol of each other (Figure S2). However, since docking poses are determined by their docking score, which is often poorly correlated with the binding affinity [10], we used all top five poses for subsequent analysis.

**Figure 1:**
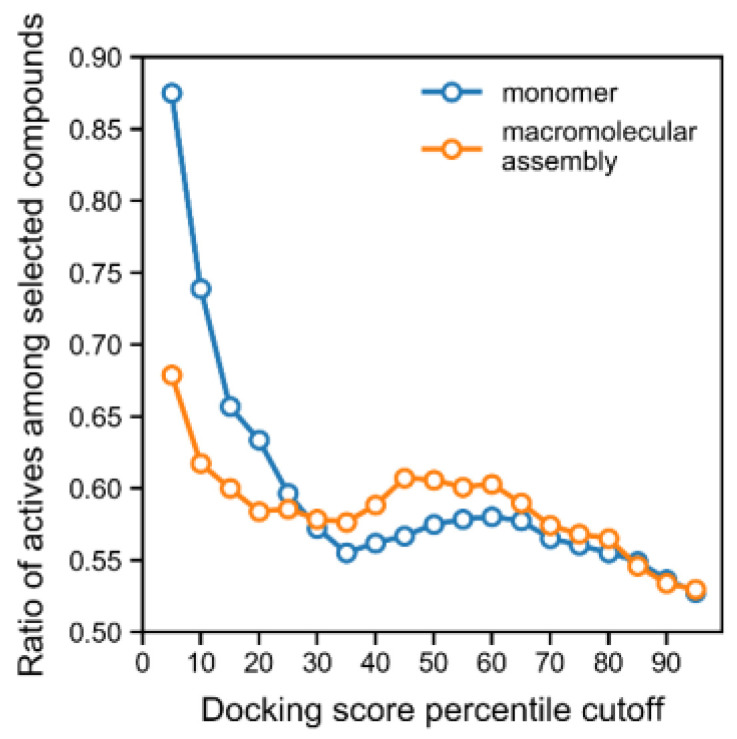
Enrichment curve of the ratio of active class vs. docking score for both the monomer (blue) and assembly of urease (orange). Our results suggested a better enrichment when the monomer was utilized, with a direct correlation between high docking score and the activity being at least as active as thiourea or acetohydroxamic acid. The data used to produce this plot correspond to the training and test sets together.

Typically, the correlation between the experimental inhibition and the docking energy is a poor surrogate for describing activity, and overall, we observed these limitations for a significant number of scaffolds produced from clustering the data. Indeed, docking scores failed to identify the majority of the most potent inhibitors (Figure S3). Nevertheless, when considering the full training set, a high docking score was a good predictor of the active class (*p* = 8.6 × 10^−19^ Spearman’s test for the correlation between score and activity), and using an increasingly lower docking score cut-off led to the enrichment of compounds from the active class (Figure 1). Indeed, all of the lowest docking scores (<−11), across all training and tests, corresponded entirely to active compounds (Figure 1 and Figure S3). Furthermore, we carried out docking on the macromolecular assembly made available in PDB (for the same PDB record, 4H9M), which consisted of a urease homodimer where the binding pocket is additionally shaped by a portion of the other monomer in the dimer. The calculations on this assembly structure interestingly did not improve the enrichment of the active class, but rather, degraded the predictive performance of the docking score, which could be observed with a smaller enrichment for the active class as a function of docking score cut-off (Figure 1). Overall, the steadily increasing enrichment of actives for the “monomer” docking calculations shown in Figure 1 shows that the docking score is a good predictor of activity and can be used as a filter in virtual screening.

The analysis of the 100 top-scoring compounds from docking revealed that these were predominantly substrate analogues and transition-state analogues as well as sulfonamides (known to be good binders of zinc). These top 100 compounds showed an average docking score of −10.86 and an average molecular weight of 527.20 g/mol (with 524.73 vs. 540.16 g/mol for active and inactive class, respectively). This small discrepancy between the averages for actives and inactives suggests that the relatively larger size of the inactive class may explain their apparently “improved” docking scores.

Even though the average activity ratio for the top 100 compounds was 2.414, the median was rather low, at 0.169, which means that the top-scoring compounds tend to be rather potent. Indeed, 84% of these compounds were actives (i.e., below a ratio of 1), 45% were under a ratio of 0.1, 13% under a ratio of 0.01 and 11% under 0.001. This means that the docking score would be theoretically able to find a compound with close to 2 μM of inhibitory activity with a 45% chance, and around 11% chance of finding one around with 200 nM of activity.

Since the docking score tends to penalize smaller molecules, there have been various approaches to cope with this bias and to allow finding more selective and potent inhibitors [11]. One common approach is to calculate the ligand efficiency (i.e., docking score divided by number of heavy atoms). However, when we used ligand efficiency to sort the compounds in our training set, we observed that its predictive power was much lower (around 55%), with a Spearman’s test showing a non-significant correlation (*p* = 0.21) even within categories of the same number of heavy atoms (Figure S3).

Considering that the docking score allowed for a good enrichment profile for the active compounds (Figure 1, monomer curve), we used this metric to predict actives. Whenever the docking score was used to filter compounds, we considered a cut-off of −9.43 kcal/mol, which corresponded to the 10th percentile of all docking scores. This cut-off was selected for being associated with an acceptable precision (0.74) when predicting actives (see Figure 1). The predictive performance of this docking model was also validated on the external set and temporal set, and the previously selected cut-off yielded a precision for the active class of 0.76 and 0.87, respectively. On the other hand, docking scores ranged between −1.98 and −11.45 kcal/mol.

Azizian et al. used docking to screen 737,685 compounds from ZINC8 which led to the selection of three compounds of different classes, one of which showed inhibitory activity and was further modified to generate barbituric acid analogues with moderate activity [12]. Similarly, docking of an in-house dataset of approximately 10,000 compounds led to the discovery of compounds with moderate activity [13]. Even though these studies proposed moderate activities, the predictive power of docking was not validated with a set of known actives, and the docking score was used with sole reliance on a good self-docking performance.

### 2.3. Protein–Ligand Interaction Fingerprints (PLIF) Identify Key Residues of Interactions

A useful way of drawing information that helps guide drug design is to analyze the protein–ligand interactions in the docking poses. Even though the docking score itself showed good predictive performance, it lacked information regarding what structural elements were contributing to the activity. This can be addressed with PLIFs, as they provide additional three-dimensional information on the types of interaction observed. If many inhibitors interact with a protein via a similar type of interaction, potentially they have similar activity. Therefore, this allows “rescuing” compounds that may have been penalized by both the scoring function of docking or missed by the machine learning model [14].

Both active and inactive classes of compounds on average interacted with a similar number of residues (5.85 ± 1.99 residues for actives vs. 5.97 ± 2.09 residues for the inactives), and similar amounts of interactions for each interaction type were observed for both classes (Table S1). Surprisingly, despite several families of inhibitors being designed to bind to the metal center, slightly more metal interactions per compound were observed for the inactive class (0.740 and 0.925, respectively).

**Figure 2:**
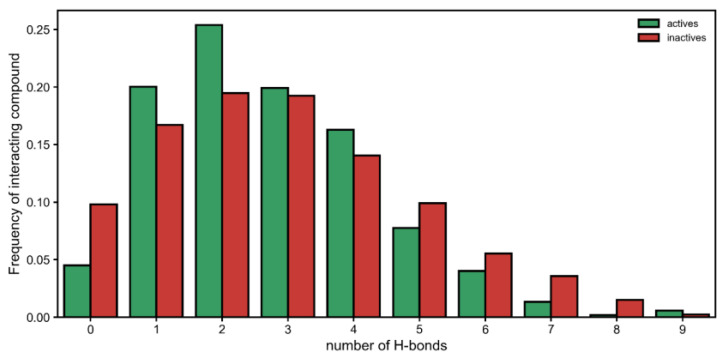
Distribution of the frequency of number of hydrogen bonds established by each compound in the active (green) and inactive (red) classes of urease inhibitors. The distribution suggests that compounds having no hydrogen bonding with urease or having five or more are more likely to belong to the inactive class. On the other hand, compounds that display one or two hydrogen bonds are more likely to belong to the active class.

Moreover, by looking at each interaction type, we observed that there was a significant difference in hydrogen bonds established by active and inactive classes (Figure 2). On one hand, the inactive compounds were more likely to have no hydrogen bonds with urease. Then, having one or two interactions seemed to be a better predictor of the active class. However, when five or more hydrogen bonds were established, this seemed to either (1) deteriorate activity (occurring more in inactives) or (2) become increasingly more irrelevant toward generating activity (Figure 2). Therefore, having many interactions via hydrogen boding might be either detrimental or at least unhelpful.

**Figure 3:**
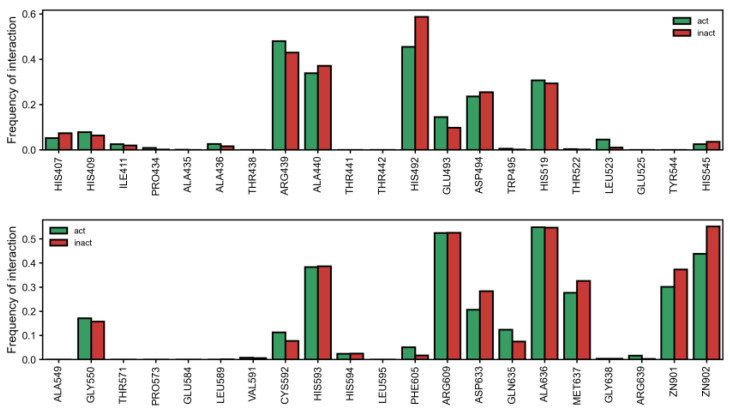
Percentage of any type of interaction in active (green) and inactive (red) classes for each urease residue. act: active, inact: inactive.

Together with the type of interaction, the types of residues that interact with compounds also appears to dictate activity in general. Surprisingly, we observed that more inactives interacted with the Zn atom (replacing Ni in the docking calculation) (Figure 3). Furthermore, we also noticed a general trend where the residues most associated with the active class were those farther from the metal center (Figure S4). However, given that a relatively large percentage of actives bind to the metal ions (~30% and ~40% to Zn901 and Zn902, respectively (Figure 3), this suggests that lengthier compounds that are able to extend from the metal center outward and establish interactions away from the Zn (Ni) atoms may be desirable. The pattern of inhibitory interaction was also not solely due to a single residue, as compounds showed a complex interaction pattern with many residues. Nevertheless, it should be noted that there were clearly residues that interacted more than others with compounds of either class (Figure 3). Besides Zn, several residues that interacted more frequently with inactive compounds were HIS492 (OR of 0.58), and to a lesser degree, ALA440 (OR = 0.87), ASP494 (OR = 0.90), ASP633 (OR = 0.65) and MET637 (OR = 0.79). Contrarily, interactions with ARG439 (OR = 1.22) were more frequently observed in the active class, which, alongside the higher percentage of pi-cation interactions, may explain their favorable profile (Table S1). Indeed, pi-cations with ARG439 were almost double in active over inactive classes of compounds. Other important residues for activity were CYS592 (OR = 1.52), GLN635 (OR = 1.74) and GLU493 (OR = 1.55).

Next, we exhaustively mined interaction patterns that were predictive of activity by evaluating all combinations of urease residues (three, four or five simultaneous residues) where at least a minimum of 70% of 40 compounds were shown to be from the active class (see Table 2). Starting with combinations of three residues, we observed the combinations of the same residues to be the most relevant to make predictions. In particular, it seems that ARG-609 is a very important residue and appears in many of these combinations to contribute to the enrichment of compounds belonging to the active class. Curiously, these combinations also pointed to the importance of ASP494. After mapping these residues in the crystal structure, we observed that ASP494 was right in front of the ARG609 and, therefore, they probably interact with one another via salt bridges, and it is likely that compounds that can insert into that site may disrupt this interaction.

Increasing the length of combinations to four residues resulted in different combinations of nearby residues but generally evidenced the positive role of ARG609, HIS519 and ASP633 in combinations associated with activity.

Further increasing the length of combinations to five revealed that the combination ALA636/ARG439/HIS492/MET637/ZN902 (combination 10) had a hit rate of 70% for 54 compounds (Table 2). Again, ARG609 was found to be important for activity, and another interesting combination found was ASP633/GLY550/HIS519 with both metal ions of the active site (combination 14). In combination with these residues, HIS593 was relevant but only in combination with ASP494 and not GLU493. This was expected since in the pocket in this residue is positioned next to ARG609 and in front of ASP494, while relatively far from GLU493.

Building a decision tree with PLIFs revealed interesting patterns that differentiated the active from inactive class. For example, interaction with HIS492, MET637, and HIS593, but not with ALA636 and ZN901, was able to enrich the number of active over inactive compounds (126:51 ratio). This rule was even more interesting when we considered that the residues at the top of the corresponding branch individually interacted more frequently with inactive compounds (Figure 3). Similarly, interaction with HIS492, MET637, not with HIS593, and with ARG-439 and GLY-638, enriched actives over inactive compounds (110:17). This highlights that even if interactions with ASP494 and MET637 are unfavorable, the interaction with ARG439 is overwhelmingly correlated with activity. For compounds that did not interact with HIS492 but interact with GLY550 and PHE605, if they then interacted with HIS593, we found a small enrichment of 102/78 for the active over inactive class, but if they did not interact with HIS593 but rather with MET637, the rule enriched with the inactive class (61 active to 122 inactive). Despite the observations that interactions with the metal centers seemed unfavorable, the decision tree algorithm found that compounds not interacting with HIS492 and GLY550 but interacting with ASP494, MET637 and ZN901 were two times more likely to be active (68/33). Even though decision trees provided interesting patterns of interaction, they performed the worst in separating active from inactive compounds compared to the combinations described above.

Overall, the PLIF analysis suggested a complex network of interactions between compounds and the jack bean urease active site. Many different combinations of residues seem to “fine tune” the interaction between a ligand and the protein and dictate inhibitory activity. In particular, a small set of residues appeared to be dominant in defining the type of interaction and activity.

### 2.4. Machine Learning Can Accurately Predict Activity of Urease Inhibitors

Many QSAR models have been developed for different classes of urease inhibitors [15,16,17]. However, achieving a general and accurate prediction model that can be applied in structure-based virtual screening is still a very demanding task. For this purpose, we built a machine learning to produce a classification model that predicts whether a compound is active or not (activity defined by the ratio against a control) using aggregated chemical information extracted from docking and general chemical features.

We used three tree-based machine learning algorithms to build models that the predict active and inactive compounds: random forest (RF), extreme gradient boosting (XGBoost) and decision tree. The three candidate models were optimized through hyperparameter tuning using the grid search function (GridSearchCV), and optimized models were then tested against the test set (performance results are summarized in Table 3). The RF model (built with 200 trees) showed the highest F1-score values for both active and inactive (0.80 vs. 0.77) and was selected as the best model. This model also outperformed the other two in terms of precision and recall, with the only exception being the recall of the inactive class, which showed the highest value in the XGBoost model.

Regarding the features that composed our model, the majority of the top features included physicochemical descriptors, and interestingly, many of these were 3D in nature: SpherocityIndex, RadiusOfGyration, Eccentricity and Asphericity (see details in RDKit’s documentation https://www.rdkit.org/docs/source/rdkit.Chem.Descriptors3D.html, accessed on 2 April 2023). Furthermore, among the top 50 most important features, we also found Morgan Fringerprint bits and PLIF bits (such as HIS492, HIS593 or ALA636) to be relevant. Both the high importance of 3D physicochemical features as well as PLIFs suggested that addressing the three-dimensionality of the binding is important and provides useful information when modelling urease inhibition. This is something that has not been accounted for by other models previously published.

**Figure 4:**
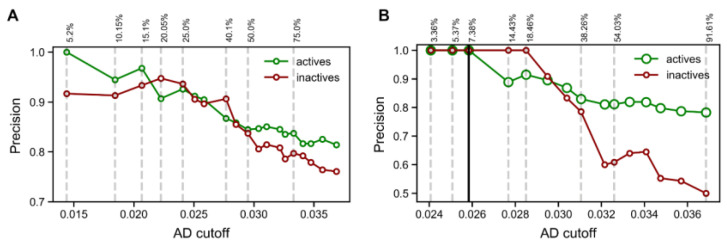
(**A**) Predictive performance measured on the test set, for different AD cut-offs. (**B**) Predictive performance measured on the external set (N = 298), for different AD cut-offs. Decreasing values of AD cut-off indicate a more stringent AD has been enforced (i.e., higher confidence in predictions is theoretically expected). The values above the plot indicate data coverage (i.e., predictions allowed by the AD cut-off).

The optimal modelling conditions for the RF model were then selected to build an ensemble of 100 RF models where varying random seeds were used for the sampling of data and features during training. The resulting 100 predictions for each compound were then aggregated using majority voting to produce the final classifications. The trained model was subsequently tested on the test validation set (Figure 4A) and the external set (Figure 4B) and showed precision similar to that of the base model for the active class. However, enhancing the prediction with an applicability domain (AD) method, this model was able to effectively filter out unreliable predictions (i.e., mispredictions). As the AD cut-off became more stringent (lower value), the precision of predictions in both active and inactive classes steadily increased. In fact, using the most stringent cut-off, it was possible to achieve 100% precision when predicting the active class. The drawback of using such a stringent cut-off is that data coverage becomes very low (i.e., only 5.2% and 7.38% of predictions were accepted to be reliable). Based on these results, in order to use the model for screening purposes, we used the reliability-density neighborhood method to established an AD cut-off that corresponded to the maximum value at which we accepted predictions with sufficient reliability. This value was selected from the AD performance curve measured in the test set (Figure 4A), where a cut-off of 0.258 was the highest value associated with ~90% precision for both active and inactive classes. This result not only validated the reliability of the predictions produced by the machine learning model but also validated the usefulness of the AD cut-off selected. Additionally, it is worth noting that both the AD curves for the test set and the external set showed an overall indirect correlation between the AD cut-off and precision of predictions. This meant that the method used to define the model’s AD has the desired relationship with prediction performance (i.e., as the cut-off becomes more stringent, prediction performance tends to increase steadily).

**Figure 5:**
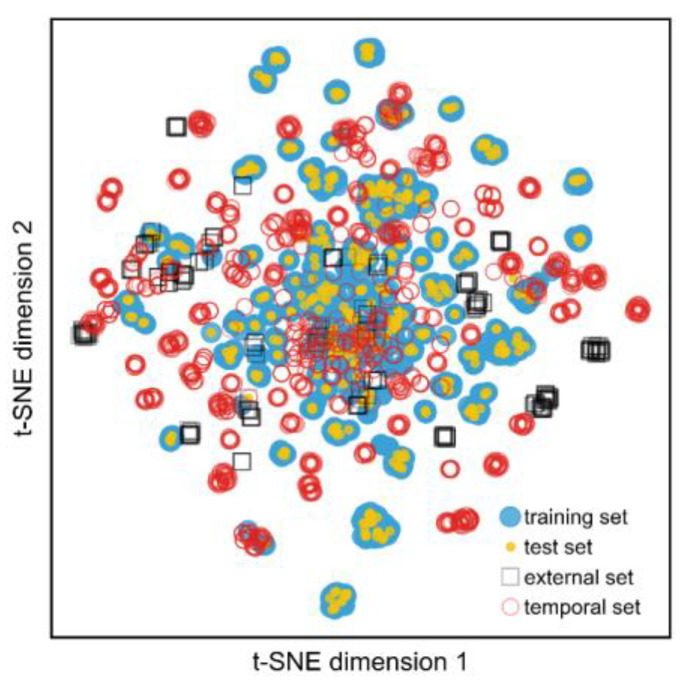
Chemical space distribution using t-SNE of the Morgan fingerprints of the different datasets. As can be observed, the temporal dataset (red) significantly drifts and expands from the original chemical space of the model, making it harder to accurately predict their activity.

To further validate our model’s predictive performance, we tested it on data published after the publications in the training set (i.e., from 2021 to 2023). After curating this dataset, we were left with 728 compounds with activity data against jack bean urease. Contrary to previous tested datasets, we observed a precision of 0.67 and recall of 0.82 for the active class. Given the passage of time and its inherent chemical space drift, a portion of these predictions were extrapolated from the chemical space covered by the model (Figure 5). Therefore, employing an AD filter was even more important in this scenario and, indeed, doing so yielded a significant increase in the precision and recall of the active class (i.e., 0.67 and 0.71, respectively). However, we should note that, in this case, an even more stringent AD (0.25) cut-off was used compared to the test set, since the original cut-off produced significantly poorer precision (0.67). This indicates that the model performance should be “recalibrated” periodically as new scaffolds are being published.

Previous in vitro screening efforts for sets of 84 and 3904 compounds were able to discover new urease inhibitors with different mechanisms of action [6,7]. Screening a large number of compounds is, however, usually costly, and for this reason, there has been an interest in predicting urease inhibition for virtual screening. Recently, using machine learning classification and regression models such as associative neural networks, k-nearest neighbors, XGBOOST, and WEKA-RF (RF model in WEKA), with cross-validation on a small dataset containing 647 compounds (518/129 split) showed the potential of predicting activity [8]. Even though the experimental compounds (thiazole based scaffold) were all weaker than thiourea (around 50 μM and, therefore, classified as inactive by our model), the predicted activity was accurate [8]. Using the combination of docking with a Monte Carlo method-based QSAR model using simplified molecular-input line-entry system (SMILES) and GRAPH descriptors on a dataset of 436 urease inhibitors from BindDB similarly achieved high prediction performance for the test sets but was not tested against an experimental validation or any external dataset [18]. Alternatively, virtual screening with the 3D shape-based Rapid Overlay of Chemical Structures Tanimoto score based on the compound *o*-chloro-hippurohydroxamic acid was used to screen the enamine library of 1.83 million compounds, of which 1700 were then docked into urease. Afterwards, eight compounds of different classes from the top 100 ranked compounds were tested in vitro, and all showed activity lower than thiourea (0.32–12.53 μM vs. 22.61 μM respectively) and were shown to be competitive and mixed-type inhibitors [9]. Even results from the virtual screening described above are promising; these models were still very limited in the number of compounds used. Based on our results, this is very important, as even after using a relatively large dataset, its accuracy dropped significantly for new compounds with distinctive scaffolds. For instance, the loss in prediction power in the temporal dataset can be attributed to the drift from the original chemical space known by the model, given that the collected data for this dataset were published after the data that made up the training and test sets. On the other hand, a limitation of using docking-based methods for urease is the assumption of “competitive” binding. However, from the reported literature that was used to assemble this dataset, compounds tested against urease were mostly mixed-type inhibitors and several uncompetitive and non-competitive inhibitors as well as covalent inhibitors that were also present. Their inclusion thus may skew the results, as their binding to the active site is not realistic, and current models do not discriminate the type of mechanism by which compounds may act.

### 2.5. Proof-of-Concept Shows the Usefulness of Our Machine Learning Model: In Vitro Urease Inhibition Assay of an in-House Library

**Figure 6:**
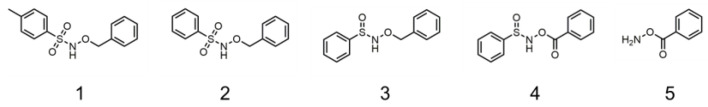
Compounds selected from virtual screening and selected for further in vitro testing.

We then screened an in-house library of 106 compounds. Molecular docking yielded no actives, but the machine learning model predicted eight actives (albeit outside of the applicability domain), from which five were visually selected for in vitro testing. The compounds predicted to be active included some sulfonamide and sulfinamide analogues (Figure 6), and these were further selected to test in vitro against jack bean urease (Table 4). Benzene sulfonamides are a well-established class of urease inhibitors, with very potent compounds having been reported [19], whereas the sulfinamide group has never been tested against urease, to the best of our knowledge. The selected compounds were, however, very closely similar to sulfonamides previously reported to have weak activity [20].

As described in Table 4, all compounds predicted by the ML model were outside the applicability domain and, therefore, the predictions should not be accepted. However, these were still tested, as they belong to an often-active scaffold. Indeed, using the AD filter would have prevented us from accepting two mispredictions. All active compounds showed moderate-to-strong activity, and compound **2** and **3** with the lowest IC_50_ were further investigated for their mechanism of inhibition by a preliminary enzyme kinetics assay using different concentrations of compounds and substrate. A Lineweaver–Burk curve was then fitted to these data, being used to provide an initial indication of the mode of inhibition. For compound **2**, the decrease in *V*_max_ and increase in *K*_M_ indicated a mixed-type inhibition (Figure S5). On the other hand, for compound **3**, it produced parallel lines in the double reciprocal plot, and both *V*_max_ and *K*_M_ decreased with increasing inhibitor concentration, suggesting an uncompetitive inhibition pattern. Even though our docking protocol is specifically meant to find competitive inhibitors, the fact that compound **3** was shown to be uncompetitive further highlights the difficulty in predictions based on this approach.

From the limited number of compounds, it appeared that the methyl group (compound **1**) was detrimental for the activity of this class of compounds. On the other hand, the sulfinamide group seemed to be significantly more potent but led to an uncompetitive type of inhibition (compound **3**). However, restricting the degrees of freedom of the compound with the introduction of a carbonyl group led to complete loss of activity (compound **4**), even though the fragment of the molecule (compound **5**) seemed to have activity similar to that of thiourea.

## 3. Materials and Methods

### 3.1. Dataset Preparation

A dataset consisting of compounds tested against jack bean urease was used from a previously assembled dataset [4]. Briefly, compounds were manually retrieved from the available literature (up to April 2021), patents and from CHEMBL28 [21]. A total of 1614 compounds tested against jack bean urease were obtained as SMILES, and only compounds with available IC_50_ or *K*_i_ values were considered. To allow better comparison between assays from different sources, the activity was normalized by being divided by the activity of the control in the corresponding assay (thiourea or acetohydroxamic acid, which are those typically reported). This is particularly important here, considering the multiple orders of magnitude of variation in IC_50_ values observed for the positive controls. Therefore, we refer to the activities in this dataset as *activity ratios* throughout this work. The compounds were then divided into active and inactive classes based on an activity ratio cut-off of 1 (actives below 1). The SMILES accompanying the activity data retrieved were standardized and cleaned using the structure preparation library MolVS 0.1.1 (https://molvs.readthedocs.io/en/latest/, accessed on 1 March 2023) in python and then converted into InChIKeys. Duplicated compounds were excluded using InChIKeys (the lowest activity value among duplicates was kept), and metal complexes were also removed.

The dataset was then divided into a training set containing 613 compounds in the active class and 597 compounds in the inactive class and a test set containing 404 compounds. Additionally, two datasets of 298 and 728 compounds, respectively, were assembled from the literature, after the final model had been built, using the methodology described earlier in this section. These two datasets were used, respectively, as an external dataset (data collection occurred after the model was finalized, but the time of the data overlaps that of the training data) and a temporal dataset (data collection happened after the model was trained, and the publication time took place after the time span of the training data) to validate our model in a way that closely emulated the real-world use of the model.

### 3.2. Similarity, Chemical Space Visualization, Molecular Descriptors and Morgan Fingerprints

The standardized structures of all urease inhibitors were used to calculate the 1024-bit Morgan fingerprints with RDKit. Additionally, we used RDKit to calculate 2D and 3D physicochemical descriptors. Visual clustering was performed with the t-SNE [22] dimensionality reduction, using the function implemented in scikit-learn (TSNE function) projecting the original 1024 dimensions into two final dimensions. Additionally, the Tanimoto coefficient value was calculated to assess the similarity within each dataset.

### 3.3. Protein Structure Preparation and Molecular Docking

For this, the X-ray crystal structure from the PDB 4H9M (1.52 Å resolution), deposited in the Protein Data Bank, was chosen, as this was the only holo structure of jack bean urease available, and it was bound to the known inhibitor acetohydroxamic acid. The protein–ligand complex underwent structure preparation in MOE v.2020, which entailed washing (removal of salts, ions, crystallography additives and solvent) while leaving the two Ni atoms in the ligand’s binding site. Next, the structure was energy-minimized through optimization of the intramolecular hydrogen bonding network within the protein (performed with Protonate3D in MOE). Additionally, the CME residue was changed back into a cysteine, as this alteration was originated during the crystallization process, while KCX (carbamylated LYS) was kept unchanged, as it naturally occurs in this protein. Finally, the Ni atoms were replaced by Zn atoms, as the parameters for the latter were already parameterized in the docking software used (LeDock [23]) and were the most similar in overall properties. Considering that PDB 4H9M was the only holo structure available, we validated our docking procedure with self-docking alone using the RMSD between top-scoring pose and the natural ligand as the evaluation metric.

The full dataset of urease inhibitors was then submitted to molecular docking calculations using LeDock. Prior to any calculations, the dataset was also prepared in MOE prior to docking calculations, where all compounds were converted into their most probable tautomeric and protomeric state at pH 7.4 and rendered into a 3D structure through energy minimization using the Amber10:EHT method. The docking box was generated for the active site of the enzyme defined from the co-crystallized ligand and extended to visually accommodate the full cavity. Compounds were then docked for a set of 1000 poses per compound. Protein–ligand interactions and docking scores were the two main outputs from the molecular docking calculations.

Additionally, the enrichment profile for the active class was also used as additional validation of docking’s predictive performance, and the docking score of both the top-scoring and the five top-scoring poses were used for prediction and analysis.

### 3.4. Protein–Ligand Interaction Fingerprints (PLIFs)

The top 5 docking score poses for each compound were used to derive protein–ligand interaction fingerprints (PLIFs) with the PLIP software v1.1.0, using the docker-based command following instructions in PLIP’s GitHub repository (https://github.com/pharmai/plip, accessed on 1 January 2023) [22].

Each compound’s PLIF was composed of 41 residues, which resulted from merging all residues that interacted with at least one compound in the entire dataset (training and test). To avoid a very sparse PLIF matrix, we opted to handle residue interactions as binary (interaction exists or not, regardless of its type) instead of representing them as residue-interaction type. Considering that multiple poses of the same compound may have distinct interaction signatures, consensus of interactions was defined as interactions being required to be present in at least 3 out of the top 5 poses.

The generated fingerprint of interactions resulted in a 1614 × 41 binary matrix that was then further analyzed using scipy and sklearn to calculate the number of interactions, combination of interactions and the odds ratio of each interaction, and finally, a decision tree model was built to extract meaningful rules associated with this protein–ligand fingerprint using the DecisionTreeClassifier function in scikit-learn.

### 3.5. Construction of a Machine Learning Model for Prediction of Urease Inhibition

The dataset was split into training and test sets (75:25% split) using clustered splitting. To do this, we submitted the full data to hierarchical clustering using the AgglomerativeClustering function in scikit-learn, applied to the Morgan fingerprints of the compounds. This produced a total of 50 clusters (number of clusters set by us), from which the training and test sets were randomly sampled [4]. After sampling, the training set was used to carry out feature selection using ReliefF’s function in skrebate, where the top 30 features were selected from each of three feature sets (2D physicochemical descriptors, Morgan fingerprints and PLIFs), and all features from 3D descriptors were kept, as they were less than 30. As a result, a total of 70 features were used for the model building stage. Three machine learning methods were tested: random forest (RF) in scikit-learn (RandomForestClassifier function), XGBoost (XGB) v1.2.0 using a “binary:logistic” learning objective, and decision tree (DT) using a min_samples_split of 10. All models were classifiers (classification of active and inactive classes). Hyperparameter tuning (RF: number of trees; XGB: learning rate, max_depth, min_child_weight, gamma) was guided by 5-fold cross validation performance. The best machine learning method was selected based on the test set performance (measured as F1-score), and then used to train an ensemble of 100 models with bootstrapping without replacement. The final class prediction was obtained by taking the majority vote from all 100 models. To assess predictive performance, the trained ensemble was tested against the external validation dataset and temporal dataset, which were composed of data that were acquired from the literature after the model had been built (see Table 1). The reliability-density neighborhood applicability domain method was used to define the models’ applicability domain [24].

### 3.6. Evaluation and Validation Criteria

In order to measure the classification performance of our model, we used the following metrics using sklearn: Precision (Equation (1)), Recall (Equation (2)) and F1-score (Equation (3)).

(1) $$Precision\;=\;\frac{True\;Predicted\;Positives}{All\;Predicted\;positives}$$

(2) $$Recall\;=\;\frac{True\;Predicted\;Positives}{All\;true\;positives}$$

(3) $${F1-score}_{}\;=\;\frac{2\times Precision\times Recall}{Precision+Recall}$$

True Predicted Positives represent the number of correctly predicted active or inactive classes, as both were calculated.

### 3.7. Chemistry

All reagents and anhydrous solvents were purchased from standard commercial vendors and used without further purification. Compounds from an in-house library were published previously [25].

### 3.8. General Procedure for Urease Inhibition Assay

The urease inhibition assay was carried out by measuring the release of ammonia based on the indophenol method described by Weatherburn [26]. A set of in-house compounds was initially screened against urease, first at a concentration of 500 μM, and active compounds were then further screened to determine the IC_50_. Briefly, a reaction mixture containing 25 μL of jack bean urease (5 U/mL), 5 μL of test compound at various concentrations, 45 μL of 20 mM urea in 0.01 M PBS buffer and 10 μL of 0.01 M PBS buffer (0.01 M) was incubated for 10 min at 30 °C in a 96-well plate. Afterwards, 40 μL of phenol reagent (1% *w*/*v* phenol and 0.005% *w*/*v* sodium nitroprusside) and 40 μL of alkali reagent (0.5% *w*/*v* NaOH and 0.1% active chloride) were added to each well. The release of ammonia was then determined by measuring the absorbance at 625 nm after 10 min, using a microplate reader (SPECTRA MAX 240). The urease inhibitory activity was calculated according to the following formula:

$$Inhibition\left(\%\right)\;=\;100-\left(\frac{OD\;test}{OD\;Control}\right)*100$$

where OD test and OD control represent the optical densities in the presence and absence of testing compound, respectively. Thiourea was used as a standard inhibitor.

The mode of inhibition was determined by monitoring the inhibition effect of various concentrations of compounds (5–100 μM) in the assay with varied substrate concentration (0.5–5 mM) using the same method above. Lineweaver–Burk plots of 1/V versus 1/[S] of the obtained results were then used to determine the type of enzyme inhibition.

### 3.9. Statistical Analysis

All assays were performed in triplicate to test the reproducibility. The results are presented as mean ± SEM. Correlations among data obtained were calculated using Spearman’s coefficient (r).

## 4. Conclusions

Inhibiting urease is a highly appealing approach that has applications in medicine and agriculture. This is evident from the large and ever-increasing number of publications where new inhibitors are screened and/or designed. Predicting the inhibitory activity of compounds against urease is, therefore, very useful, particularly through a data-driven approach. However, to date, only small models, often built from low-diversity datasets, have been reported. In this work, we sought to bridge this gap in the literature. To achieve this, we first collected the largest publicly available dataset of compounds tested against jack bean urease and carried out extensive computational analysis of features that characterized active compounds and distinguished them from inactives. Various features, including docking score, type and number of interactions with the active as well as 3D physicochemical features of the compounds, were found to be correlated with activity. Exhaustive mining of combinations of residues revealed interesting patterns that differentiated actives from inactives, such as the observation that actives seemed to bind further away from the metal center. Additionally, the docking score correlated closely with activity and showed good predictive power when validated against the external and temporal sets. These encouraging findings prompted us to further integrate these results into a classifier using machine learning models based on decision trees. Accurate and robust predictions were obtained using random forest and were validated against various assembled datasets external to the one used to generate the model. The precision of the model was also dependent on an applicability domain filter that was used in order to exclude likely mispredictions. Moreover, the precision of the model dropped for newly reported scaffolds due to their chemical differences in comparison to the scaffolds used to build the model. The final model was then used to screen an in-house library and was able to identify new compounds with inhibitory activity in vitro. The study shows that employing a data-driven machine learning algorithm can lead to the identification of new chemical compounds against urease.

## Figures and Tables

**Table 1:** Description of the datasets of compounds tested against jack bean urease that were used to construct and validate the machine learning model. Tanimoto mean was calculated as the average of pair-wise Tanimoto coefficients among all compounds in each set.

Dataset	Total Compounds	Number of Active Class Compounds	Number of Inactive Class Compounds	Tanimoto Mean
Training Set	1210	613	597	0.13
Test Set	404	216	188	0.13
External Validation Set	298	215	83	0.15
Temporal Validation Set	728	450	278	0.13

**Table 2:** Combination of residues where at least 70% of compounds are from the active (i.e., minimum 70% hit rate) class and occur in at least in 40 compounds.

Combination	Residues in the Combination
1	ALA636, ASP494, GLU493
2	ARG609, ASP494, LEU523
3	ARG609, ASP494, PHE605
4	ARG609, HIS593, PHE605
5	GLY550, HIS492, MET637
6	ALA636, ARG609, ASP494, GLU493
7	ARG439, ARG609, HIS519, MET637
8	ARG609, ASP633, HIS492, MET637
9	ASP633, GLY550, HIS519, ZN901
10	ALA636, ARG439, HIS492, MET637, ZN902
11	ARG439, ARG609, HIS492, HIS519, MET637
12	ARG439, ARG609, HIS519, MET637, ZN901
13	ARG439, ARG609, HIS519, MET637, ZN902
14	ASP633, GLY550, HIS519, ZN901, ZN902

**Table 3:** Performance measured as precision, recall and F1-score on the test set for the three candidate machine learning models. Value in bold indicate the best performance for each metric.

	Precision		Recall		F1-Score	
	Actives	Inactives	Actives	Inactives	Actives	Inactives
Random Forest	**0.81**	**0.77**	**0.79**	0.78	**0.80**	**0.77**
XGBoost	0.71	0.72	0.73	**0.80**	0.77	0.76
Decision Tree	0.74	0.72	0.76	0.69	0.75	0.70

**Table 4:** Shortlisted compounds among virtual screening hits accompanied by their predictions from both in silico models, inhibitory activity and mode of inhibition from in vitro testing against jack bean urease.

Compounds	Docking Score * (Kcal/mol)	Docking Prediction	ML Prediction	% at 1 mM or IC_50_ ± SEM (μM)	Mode of Inhibition
**1**	−5.92	Inactive	Active	43.85%	--
**2**	−5.70	Inactive	Active	100.12 ± 10.18	Mixed-type
**3**	−5.96	Inactive	Inactive	6.15 ± 0.99	Uncompetitive
**4**	−5.48	Inactive	Active	0%	--
**5**	−6.42	Inactive	Inactive	155.19 ± 2.02	--
Thiourea	−3.48	Inactive	--	144.37 ± 5.08	--

* Docking score refers to the score from the top-scoring pose.

## Data Availability

Data is available upon request.

## Supplementary Materials

The following supporting information can be downloaded at: https://www.mdpi.com/article/10.3390/ijms24098180/s1.
