# Supplementary material for: Using Machine Learning and Molecular Docking to Leverage Urease Inhibition Data for Virtual Screening
Source: Int J Mol Sci. 2023 May 3;24(9):8180. doi: 10.3390/ijms24098180 (PMC10179503; doi:10.3390/ijms24098180)
Supplement: Supplementary file 1 [file ijms-24-08180-s001.zip › ijms-2363564-supplementary.pdf]

Supplementary Materials: Using Machine Learning and Molecular Docking to Leverage Urease Inhibition Data for Virtual Screening

Natália Aniceto [1,2], Tânia S. Albuquerque [3], Vasco D. B. Bonifácio [3,4], Rita C. Guedes [1,2] and Nuno Martinho [3,*]

[1] Research Institute for Medicines (iMed.ULisboa), Faculty of Pharmacy, Universidade de Lisboa, 1649-003 Lisbon, Portugal

[2] Department of Pharmaceutical Sciences and Medicines, Faculty of Pharmacy, Universidade de Lisboa, 1649-003 Lisbon, Portugal

[3] iBB—Institute for Bioengineering and Biosciences, and Associate Laboratory i4HB—Institute for Health and Bioeconomy at Instituto Superior Técnico, Universidade de Lisboa, Av. Rovisco Pais, 1049-001 Lisboa, Portugal

[4] Bioengineering Department, Instituto Superior Técnico, Universidade de Lisboa, Av. Rovisco Pais, 1049-001 Lisboa, Portugal

* Correspondence: nunomartinho@tecnico.ulisboa.pt

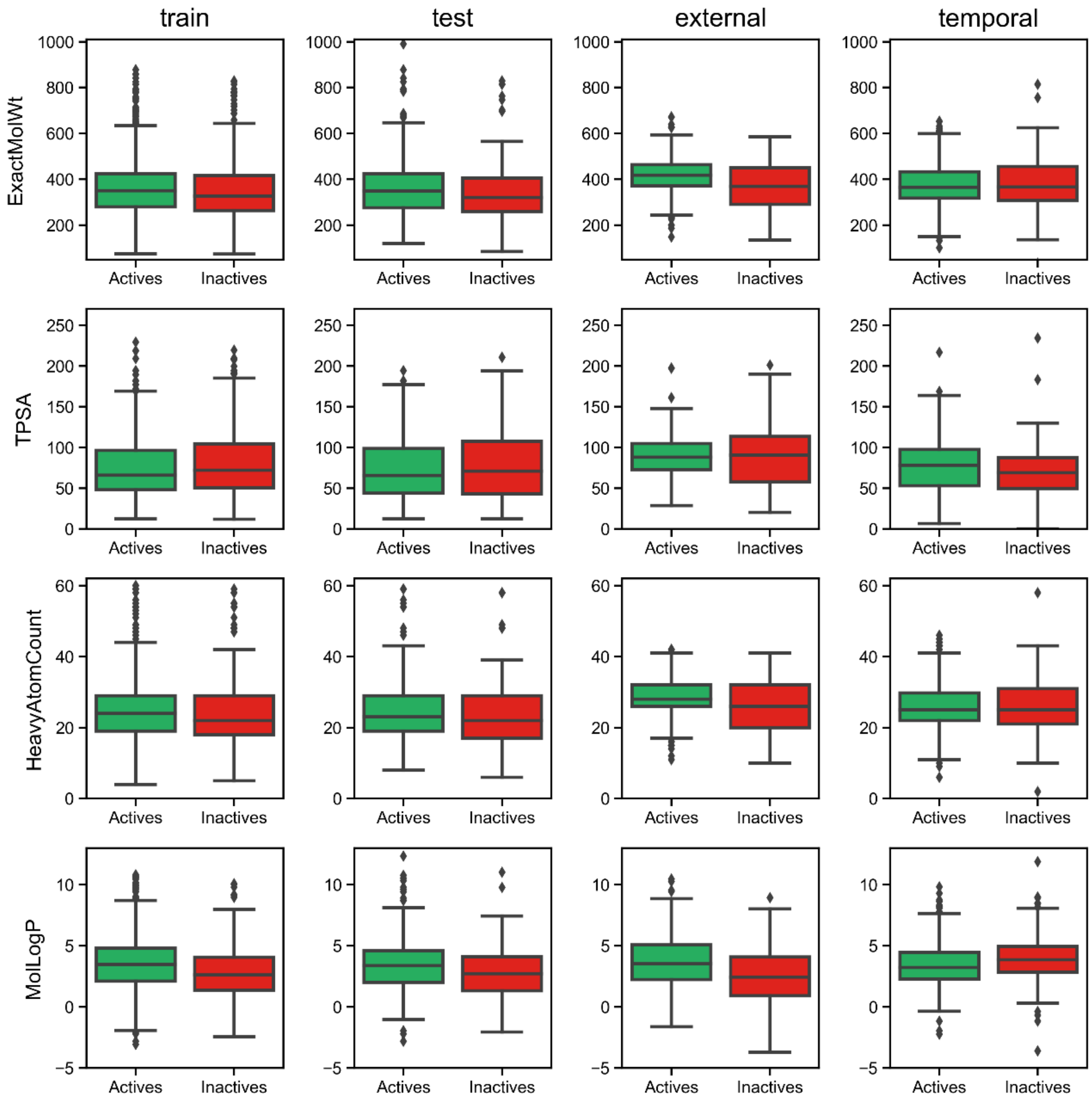

Figure S1. Distribution of chemical properties of the different dataset tested between active (green) and inactive (red) classes.

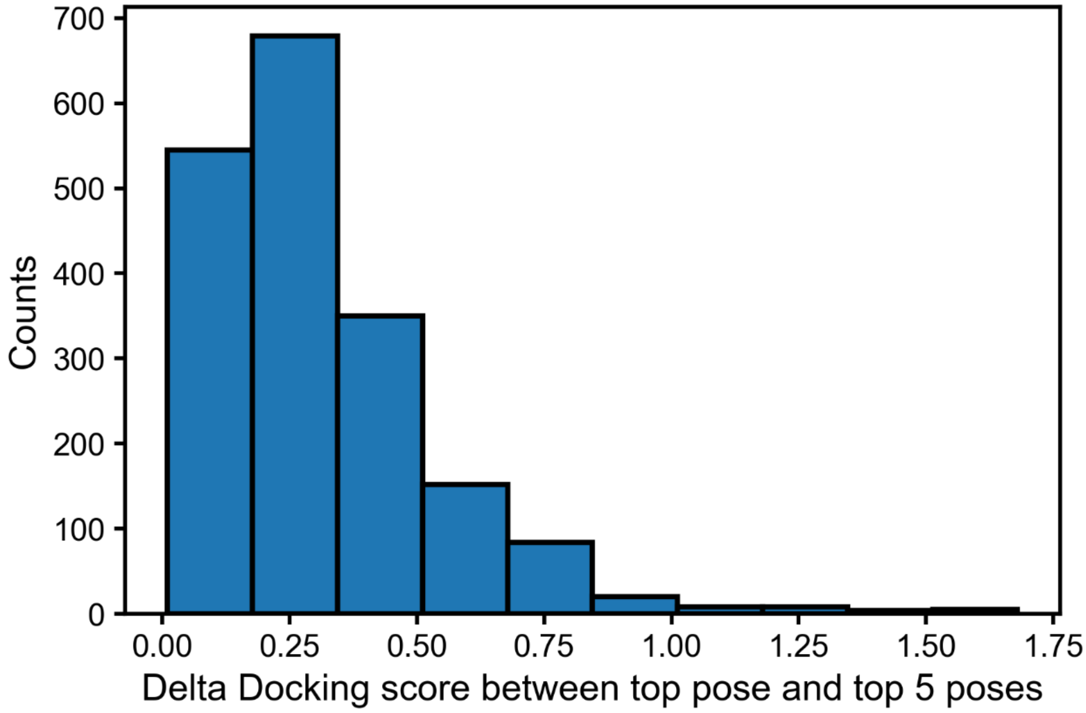

Figure S2. Distribution of the variation of docking scores for the best 5 poses.

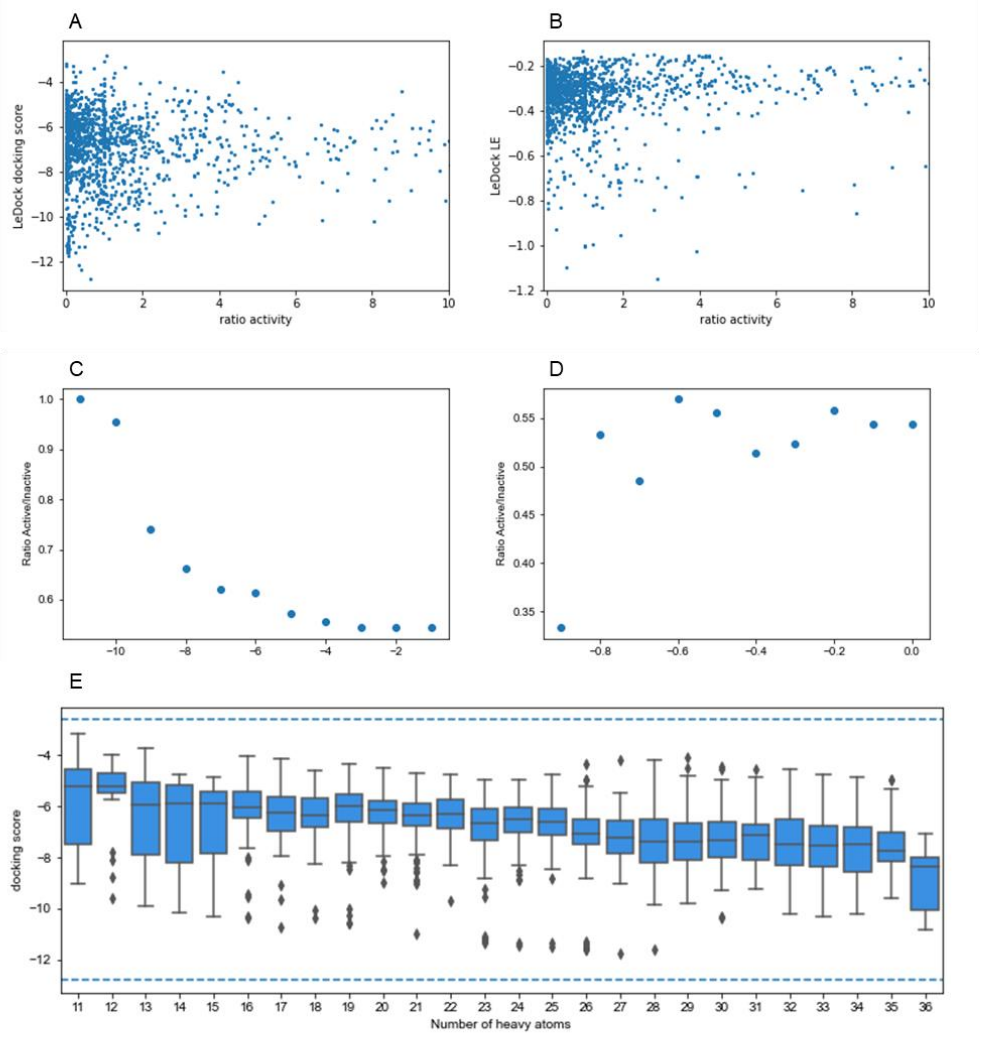

Figure S3. A) Docking score distribution as a function of activity of compound compared to control thiourea or acetohydroxamic acid; B) ligand efficiency normalized docking score as a function of activity ratio to remove effects of molecular weight in the docking score; C) Correlation of docking score with probability of finding an active class compound; D) No correlation was found for ligand efficiency normalized docking score; E) Docking Score distribution as a function of the number of heavy atoms where no significant correlation was found.

Table S1. Average number of interactions by type of interaction

	Average number of interactions per compound	
**Type interaction**	**Active class**	**Inactive class**
Hydrophobic	1.249	1.179
Hydrogen Bonds	2.747	2.914
Pi-cation	0.306	0.237
Metal	0.740	0.925
Salt Bridges	0.125	0.192
Pi-stacking	0.169	0.142
Halogen bonds	0.044	0.030

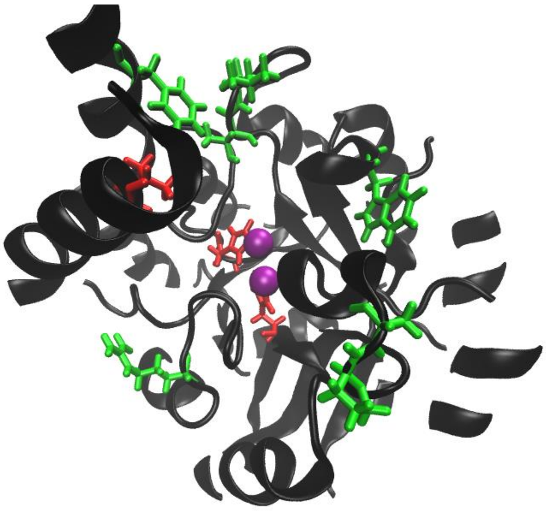

Figure S4. Fold activity (green) and inactivity (red) residues Pink represents the metal centre ions. The highlighted green residues are ALA-435, PRO-434, ARG-639, LEU-523, TRP-495, PHE-605 and THR-522. The highlighted red residues include LEU-589, HIS-545 and HIS-407.

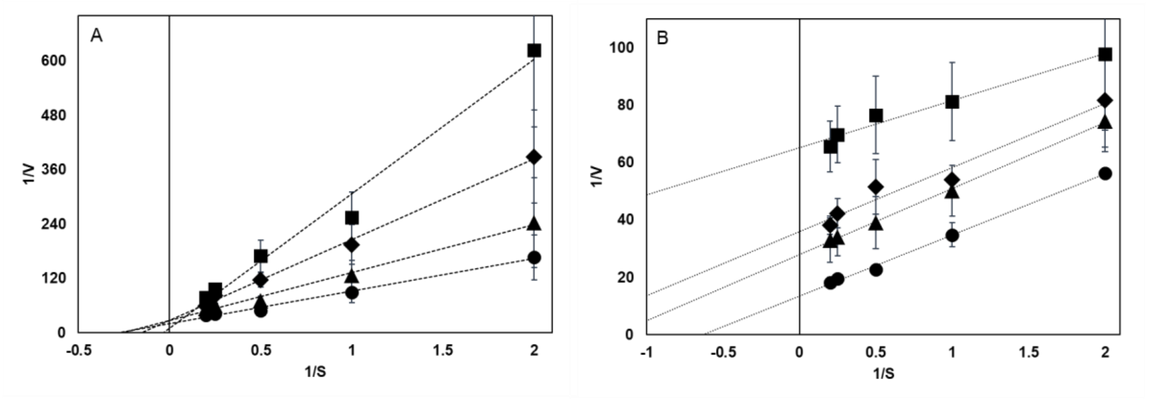

Figure S5. Lineweaver-Burk plots for enzymatic kinetics of the reciprocal of reaction rate vs the reciprocal of the substrate (urea) in the absence and presence of 0.5-100 μM of A) compound 2 (mixed-type inhibition) and B) compound 3 (uncompetitive).
